# Preoperative hydrocephalus and the risk of postoperative speech impairment following posterior fossa tumour surgery in children: results from a prospective, multinational cohort study

**DOI:** 10.1007/s00381-026-07132-z

**Published:** 2026-02-04

**Authors:** Aske Foldbjerg Laustsen, Radek Frič, Jonathan Kjær Grønbæk, Vladimír Beneš, Vicente Santa-Maria Lopez, Ulf Nestler, Andrea Carai, Guirish Solanki, Shivaram Avula, Conor Malluci, Pelle Nilsson, Per Nyman, Magnus Aasved Hjort, Rick Brandsma, Eelco Hoving, Antonella Bua, Jana Táborská, Katalin Mudra, Markia Balázs, Giedre Rutkaiskiene, Saulius Ročka, Jurgen Lemiere, Florian Wilhelmy, Christian Dorfer, Astrid Sehested, Marianne Juhler, René Mathiasen

**Affiliations:** 1 Department of Neurosurgery, Rigshospitalet, Copenhagen, Denmark; 2 Department of Pediatrics and Adolescent Medicine, Rigshospitalet, Copenhagen, Denmark; 3 Department of Neurosurgery, University Hospital Oslo-Rikshospitalet, Oslo, Norway; 4 Department of Neurosurgery, 2nd Medical Faculty and Motol University Hospital, Prague, Czechia; 5 Neuro-Oncology Unit, Pediatric Cancer Center Barcelona, Hospital Sant Joan de Déu, Barcelona, Spain; 6 Department of Neurosurgery, Fondation Lenval Children’s Hospital, Nice, France; 7 Department of Neurosurgery, Bambino Gesù Children’s Hospital, Rome, Italy; 8 Department of Paediatric Neurosurgery, Birmingham Children’s Hospital NHS Foundation Trust, Birmingham, UK; 9 Department of Radiology, Alder Hey Children’s NHS Foundation, Liverpool, UK; 10 Department of Neurosurgery, Alder Hey Children’s NHS Trust, Liverpool, UK; 11 Department of Medical Sciences/Neurosurgery, Uppsala University, Uppsala University Hospital, Uppsala, Sweden; 12 Crown Princess Victoria Children’s Hospital, Department of Biomedical and Clinical Sciences, Linköping University, Linköping, Sweden; 13 Department of Pediatric Hematology and Oncology, St. Olavs Hospital, Trondheim, Norway; 14 Princess Maxima Center for Pediatric Oncology, Utrecht, Netherlands; 15 2nd, Department of Pediatrics, Semmelweis University, Budapest, Hungary; 16 Department of Pediatrics, Lithuanian University of Health Science, Kaunas, Lithuania; 17 Clinic of Neurology and Neurosurgery, Faculty of Medicine, Vilnius University, Vilnius, Lithuania; 18 Pediatric Oncology, Department of Oncology, KU Leuven, Louvain, Belgium; 19 Department of Neurosurgery, University Hospital Leipzig, Leipzig, Germany; 20 Department of Neurosurgery, Medical University of Vienna, Vienna, Austria; 21 Department of Neurosurgery, Aarhus University Hospital, Aarhus, Denmark

**Keywords:** Cerebellar mutism syndrome, Posterior fossa syndrome, Preoperative hydrocephalus, Paediatric posterior fossa tumour

## Abstract

**Background:**

Cerebellar mutism syndrome (CMS) is a common complication of paediatric posterior fossa (PF) tumour surgery, with postoperative speech impairment (POSI) as the cardinal symptom. Preoperative hydrocephalus (pHC) is present in up to 70% of cases of paediatric PF tumours, but its association with POSI remains unclear. This study investigated whether pHC is an independent risk factor for POSI and assessed the impact of alleviating pHC prior to tumour resection on POSI risk.

**Methods:**

We included 800 children who underwent PF tumour surgery between 2014 and 2024 at 35 centres across 13 countries in the European CMS study. Speech and neurological assessments were conducted pre- and postoperatively. Neurosurgeons assessed pHC status, pHC treatment and tumour location; histology was recorded at a 2-month follow-up. pHC treatment was categorised as “yes” (pHC alleviated prior to tumour surgery) and “no” (pHC alleviated by tumour surgery alone). POSI was categorised as “habitual speech”, “reduced speech” or “mutism”.

**Results:**

Of 800 patients, 515 (64%) had pHC. Absence of pHC was associated with lower POSI risk in univariate analysis (OR 0.51 (95% CI 0.35; 0.76)), but this reversed and became non-significant after adjustment (1.20 (0.60; 2.41)). pHC treatment was associated with an increased POSI risk in the univariate analysis (1.93 (1.14; 3.26)), which became non-significant in the adjusted analysis (1.15 (0.60; 2.21)).

**Conclusion:**

The presence of pHC was not independently associated with POSI nor did treatment of pHC prior to tumour resection appear to reduce the risk of POSI. These findings highlight the importance of individualizing pHC management in paediatric PF tumour cases, with decisions guided by the clinical context.

**Trial registration:**

Clinical Trials ID NCT02300766 (October 2014).

**Supplementary Information:**

The online version contains supplementary material available at 10.1007/s00381-026-07132-z.

## Introduction

The cerebellar mutism syndrome (CMS) is primarily described as a complication of posterior fossa (PF) tumour surgery in children. Up to 30% of children are affected, but the incidence depends on the tumour location, histology and age of the child [1, 2]. CMS is characterized by a delayed onset of typically 1–10 days postoperatively, with most symptoms being transient and with spontaneous recovery in the majority of cases. It consists of four domains: (1) postoperative speech impairment (POSI), being the cardinal symptom; (2) emotional disturbances; (3) ataxia and (4) hypotonia [3]. POSI is classified into (1) reduced speech, limited to single words or short sentences, only elicited by vigorous stimulation, and (2) mutism [4].

The identification of modifiable risk factors for CMS remains a challenge. While some studies have reported an association between preoperative hydrocephalus (pHC) and increased risk of CMS [5–7], others have not found any supporting evidence [1, 8, 9], resulting in conflicting conclusions.

pHC is frequently associated with paediatric PF tumours, as the majority of these tumours present with signs of hydrocephalus at the time of diagnosis [10, 11]. Symptoms of raised intracranial pressure (ICP) often manifest from days to months before tumour diagnosis. Several objective measures for paediatric hydrocephalus, such as the frontal-occipital horn ratio (FOHR)[12], have been proposed for quantifying the severity of pHC. However, there is currently no consensus on standardised objective criteria. As a result, the diagnosis of pHC in a paediatric neuro-oncological setting relies on a combination of clinical symptoms and neuroradiological findings, based mainly on the experience of the attending physician.

The presence of hydrocephalus may increase the vulnerability of periventricular and periaqueductal structures that are involved in the pathophysiology of CMS and, subsequently, POSI. Theoretical mechanisms include direct mechanical stress from elevated cerebrospinal fluid (CSF) pressure, transependymal oedema resulting from disruption of the ependymal lining [13], and treatment-related brain shift due to the rapid release of CSF during tumour resection [14]. Therefore, early treatment of pHC in paediatric PF tumour resection could reduce the risk of POSI by eliminating the impact of increased ICP on the PF structures. Conversely, treating pHC prior to tumour resection may increase the risk of postoperative cerebellar dysfunction including POSI through physiological changes, such as alterations in regional cerebral and cerebellar blood flow, CSF clearance dynamics or inflammatory signalling, rendering increased susceptibility to surgical injury. However, these theoretical mechanisms remain speculative and require further research.

It is essential to determine whether there is a relationship between pHC and its management and the risk of developing POSI, as this may influence the treatment strategy in affected patients. Thus, our study aims are to clarify the role of pHC in the onset of POSI as well as the potential benefits of early intervention, with the following hypotheses: (1) patients with pHC are at a higher risk of developing POSI following PF tumour surgery compared to those without pHC and (2) management of pHC before tumour resection can reduce the risk of POSI by alleviating the negative effects of raised ICP on vulnerable PF structures.

## Material and methods

### Study design

The *European study on cerebellar mutism syndrome in children with posterior fossa tumours* is a prospective, observational multicentre cohort study investigating various aspects of CMS. The study received ethical approval from the Research Ethics Committee of the Capital Region of Denmark (H-6–2014-002), and its design has been published previously [15]. Between 2014 and 2024, children under 18 undergoing open surgery of a PF tumour were enrolled from 35 centres across 13 countries. Informed consent was obtained prior to inclusion or, in emergency cases, within 7 days postoperatively.

### Data collection

All patient data were collected using a structured case report form. Baseline data, including age, were recorded at inclusion. Speech and neurological assessments were conducted preoperatively and 1–4 weeks postoperatively by a neurosurgeon or paediatrician. Within 72 h of surgery, the operating neurosurgeon documented surgical details, including tumour location and the presence of pHC, recorded as a binary variable (yes/no) based on clinical and neuroradiological signs. If pHC was registered by the operating neurosurgeon, preoperative treatment modality and date of treatment were also registered (Supplementary Fig. 1). No quantitative neuroradiological assessment of pHC was included. Tumour location was recorded as one or more of the following: brainstem, fourth ventricle, cerebellar vermis, right hemisphere or left hemisphere. Tumour pathology was recorded within two months and categorised as pilocytic astrocytoma (PA), medulloblastoma (MB), ependymoma (EP), atypical teratoid/rhabdoid tumour (AT/RT) or other (Supplementary Table 1). All data were stored in a secure online database.

### Stratification of parameters

POSI was assessed as “mutism” or “reduced speech” during the follow-up period. If neither was reported, speech was considered habitual. POSI was treated as a hierarchical ordinal outcome with 3 levels (mutism, reduced speech and habitual), as this approach assumes that mutism and reduced speech are ordered levels of severity within the same condition. pHC treatment was treated as a dichotomous outcome categorised into either “yes” (pHC alleviated prior to tumour surgery) or “no” (pHC alleviated by tumour surgery alone). An additional parameter included in a supplementary analysis described the timing of pHC treatment; days from pHC treatment to tumour surgery were stratified into 5 ordered levels: “none” (pHC alleviated by tumour resection), 1, 2, 3 and ≥ 4 days. pHC treatment > 14 days prior to surgery was considered equivalent to not having pHC; four outliers were excluded on this basis (Supplementary Table 2). Tumour location, assessed by the operating neurosurgeon, was categorised hierarchically by assumed POSI risk [13]: (1) brainstem involvement (± fourth ventricle, vermis, hemisphere), (2) fourth ventricle without brainstem involvement (± vermis, hemisphere), (3) vermis only (± hemisphere) and (4) hemisphere only.

### Statistical analyses

We applied proportional odds logistic regression to estimate odds ratios (OR) with 95% confidence intervals (CI), and the proportional odds assumption was assessed using the Brant test (Supplementary Tables 3 and 4).

In the primary analysis, the presence of pHC was treated as the main binary exposure. We first performed a univariate analysis including only pHC as the explanatory variable. This was followed by a stepwise multivariate approach to sequentially adjust for potential confounders: tumour location (model 1), tumour histology (model 2) and age as a continuous variable (model 3). The final model (model 3) was considered fully adjusted and forms the basis of the main results presented in the manuscript.

A sensitivity analysis was performed by additionally adjusting for the country-level effect, which did not alter the conclusion. Furthermore, an interaction term between pHC and tumour histology was included in model 3 to assess whether the effect of pHC on POSI differed by tumour type.

In the secondary analysis on the subcohort with pHC, the impact of pHC alleviation was investigated. This analysis followed the same univariate and stepwise multivariate structure as the primary analysis, adjusting for tumour location, tumour histology and age. An additional supplementary analysis assessed the effect of pHC treatment timing and is only briefly mentioned in the main results as an exploratory analysis.

#### Missing data

The impact of missing data was assessed descriptively by comparing baseline characteristics of the overall cohort with sub cohorts used in the primary and secondary analyses to evaluate potential selection bias due to missing data (Supplementary Tables 5 and 6). Data analyses were conducted as complete case analyses. To assess the impact of missing data on model estimates, a stepwise exclusion approach was applied: at each step of the model adjustment, patients with missing values for the covariate added in the next model were excluded. This allowed for an assessment of the impact of specific subsets of missing data on the OR for the main exposure (Supplementary Tables 7 and 8). No imputation of missing data was done.

All results were reported as OR with 95% CI. Analyses were performed in R-studio (v.2024.04.2).

## Results

**Fig. 1:**
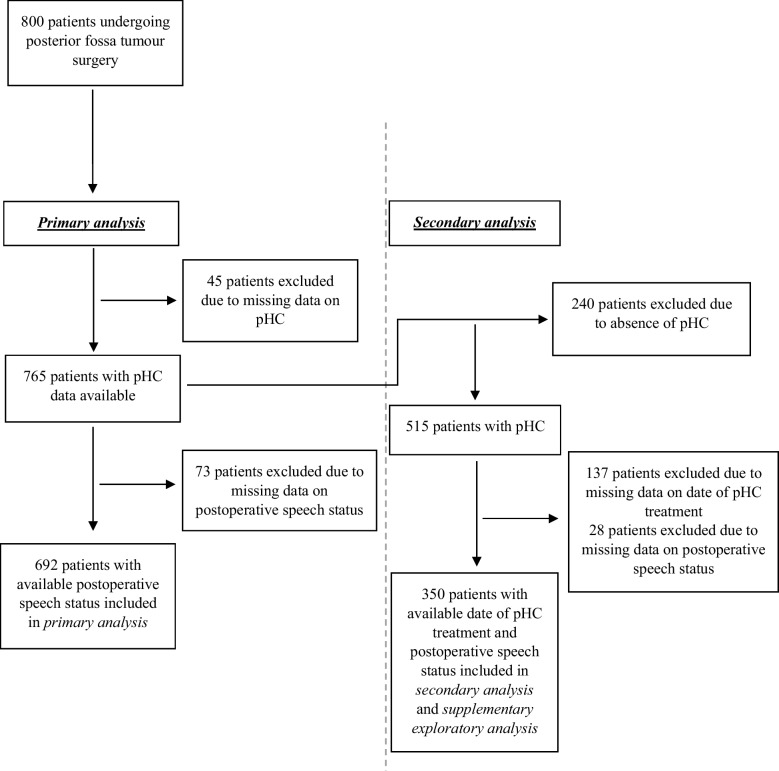
Flow chart of patients included in the primary and secondary analysis

In the period between 2014 and 2024, we included a total of 800 patients undergoing PF tumour surgery. Six hundred ninety-two patients were included in the primary analysis, and 350 patients in the secondary analysis (Fig. 1). Demographics of the cohort are summarised in Table 1. The overall cohort had a median age of 7.0 (IQR 3.9–10.9), while the subcohort included in the secondary analysis had a median age of 6.6 (IQR 3.4–10.2).

**Table 1:** Cohort demographics

	All patients (*n* = 800); *N* (% of total (vertical))	Habitual speech (*n* = 543 (68%)); *N* (% of total in the same category (horizontal))	Reduced speech (*n* = 96 (12%)); *N* (% of total in the same category (horizontal))	Mutism (*n* = 88 (11%)); *N* (% of total in the same category (horizontal))
Sex				
Male	456 (57)	307 (67)	57 (13)	48 (11)
Female	344 (43)	236 (69)	39 (11)	40 (12)
Age (years; median and IQR)	6.9 (3.9;10.9)	7.7 (4.4;11.4)	6.3 (4.1;8.9)	4.5 (2.4;8.6)
Tumour location^a^				
Brainstem	156 (20)	90 (58)	25 (16)	27 (17)
4th ventricle	265 (33)	146 (55)	42 (16)	52 (20)
Vermis	141 (18)	111 (79)	15 (11)	3 (2)
Cerebellar hemisphere	187 (23)	163 (87)	11 (6)	2 (1)
Unknown	51 (6)	33 (65)	3 (3)	4 (5)
Tumour histology				
Pilocytic astrocytoma	305 (38)	254 (83)	27 (9)	16 (5)
Medulloblastoma	222 (28)	129 (58)	40 (18)	41 (18)
Ependymoma	79 (10)	48 (61)	10 (13)	15 (19)
AT/RT	20 (2)	8 (40)	4 (20)	5 (25)
Other	73 (9)	57 (78)	8 (11)	6 (8)
Unknown	101 (13)	47 (47)	7 (7)	5 (5)
Preoperative hydrocephalus				
Yes	515 (64)	333 (65)	68 (13)	71 (14)
No	240 (30)	180 (75)	27 (11)	13 (5)
Unknown	45 (6)	30 (67)	1 (1)	4 (5)
Treatment for preoperative hydrocephalus prior to tumour surgery				
	*n* = 515	*n* = 254	*n* = 51	*n* = 45
Yes^b^	87 (17)	47 (54)	14 (16)	15 (17)
No^c^	291 (57)	207 (71)	37 (13)	30 (10)
Unknown	137 (26)			

^a^(1) brainstem involvement (± fourth ventricle, vermis, hemisphere), (2) fourth ventricle without brainstem involvement (± vermis/hemisphere), (3) vermis only (± hemisphere) and (4) hemisphere only; ^b^range 1–10 days; ^c^preoperative hydrocephalus alleviated by tumour resection alone.

PA was the most frequent tumour type (305/800 (38%)), followed by MB (222/800 (28%)), EP (79/800 (10%)) and AT/RT (20/800 (2%)). pHC was seen in 515/800 (64%), whereas 240/800 (30%) did not have pHC, and 45/800 (6%) had unknown pHC status. The predominant tumour location was the 4th ventricle (265/800 (33%)), followed by the cerebellar hemisphere (187/800 (23%)), brainstem (156/800 (20%)) and vermis (141/800 (18%)). 51/800 (6%) had unknown tumour location.

A total of 184/800 (23%) patients developed POSI: 96 patients (12%) suffered from reduced speech and 88 from mutism (11%) postoperatively. In the groups with POSI, MB was the most frequent tumour (reduced speech: *n* = 40, mutism: *n* = 41).

Of the 515 children with pHC, hydrocephalus was resolved with tumour resection alone in 291 (58%) patients, while 87 patients (17%) were treated for pHC prior to primary tumour surgery, and 137 (27%) were treated for pHC at an unknown time. Of the patients with known preoperative treatment date, 4/87 (5%) were treated with ventriculoperitoneal shunt, 27/87 (31%) with third ventriculostomy and 56/87 (61%) with external ventricular drain. No substantial differences in baseline characteristics were found between the overall cohort (*n* = 800) and the subcohort (*n* = 515) with pHC (Supplementary Table 6). The range of treatment days within the group treated for pHC prior to tumour surgery was 1–10 days, with a median of 3 days.

### Results of primary analysis

**Fig. 2:**
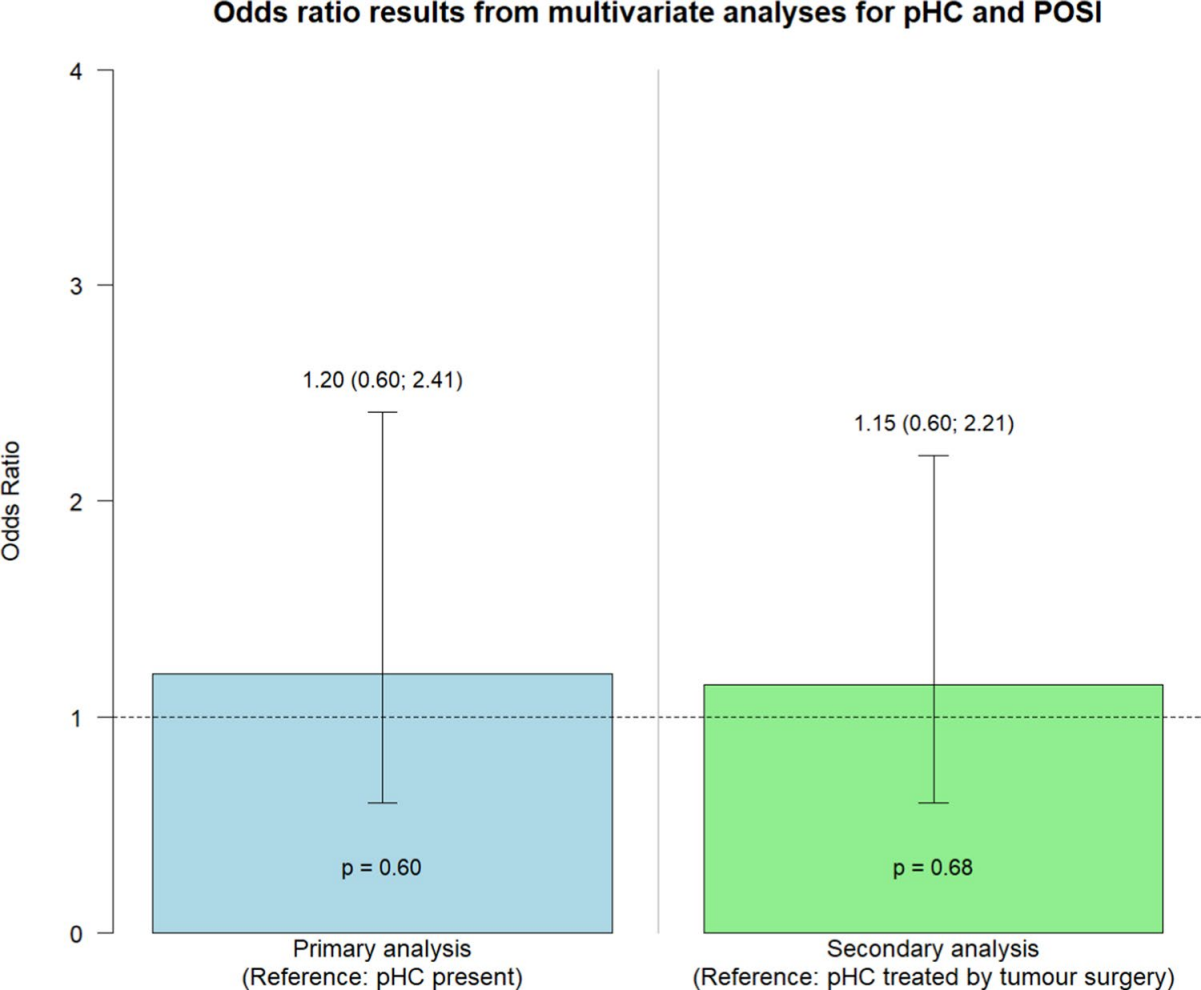
Risk of postoperative speech impairment by (1) the presence of preoperative hydrocephalus (*column 1: primary analysis*) and (2) preoperative treatment of hydrocephalus relative to tumour surgery (*column 2: secondary analysis*). pHC, preoperative hydrocephalus; POSI, postoperative speech impairment

Table 2 presents the results of the primary analysis, including the univariate analysis (column 1) and stepwise multivariate models: model 1 (column 2) adjusted for tumour location, model 2 (column 3) additionally adjusted for tumour type, and model 3 (column 4) further adjusted for age. In the univariate analysis, absence of pHC was initially associated with lower odds of POSI (OR 0.51, 95% CI 0.35; 0.76), but this association was not upheld in the final multivariate model (OR 1.20, 95% CI 0.60; 2.41) (Fig. 2). We have previously determined that MB tumour type, tumour location in the brainstem or 4th ventricle and younger age are statistically significant factors for the risk of POSI [4, 16]. These statistical relationships were confirmed by re-calculated statistics in the present study based on combining the previously published cohort (*n* = 500) and patients included subsequently (*n* = 300) (Supplementary Table 7). The tumour-dependent effect of pHC on the risk of POSI did not significantly vary by tumour pathology (Table 3).

**Table 2:** Odds ratio of postoperative speech impairment

Univariate analysis		Multivariate analyses		
		*Model 1 (tumour location)* *n* = *666*	*Model 2 (model 1* + *tumour type)* *n* = *616*	*Model 3 (model 2* + *age)* *n* = *614*
	*n* = *692*			
Preoperative hydrocephalus *Reference: yes*				
No	0.51 (0.35; 0.76) *p* < *0.001*	0.62 (0.41; 0.95)	1.11 (0.56; 2.21)	1.20 (0.60; 2.41) *p* = *0.60*

Odds ratio results with 95% confidence intervals; *n* patients included in model

**Table 3:** Interaction between pHC and tumour type on risk of POSI (model 3)

Tumour type *Reference: interaction between pHC and PA*	
MB	0.58 (0.21; 1.62), *NS*
EP	0.19 (0.03; 1.06), *NS*
AT/RT	1.05 (0.09; 11.98), *NS*
Other	0.52 (0.12; 2.21), *NS*

Odds ratio results with 95% confidence intervals, *PA* pilocytic astrocytoma, *MB* medulloblastoma, *EP* ependymoma, *AT/RT* atypical teratoid/rhabdoid tumour, *pHC* preoperative hydrocephalus, *NS* not significant

### Results of secondary analysis

Table 4 shows the results of the secondary analysis, including the univariate analysis (column 1) and the stepwise multivariate models for the subcohort with preoperative hydrocephalus: model 1 (column 2) adjusted for tumour location, model 2 (column 3) additionally adjusted for tumour type, and model 3 (column 4) further adjusted for age. In the univariate analysis, pHC treatment was significantly associated with an increased risk of POSI (OR 1.93, 95% CI 1.14; 3.26). However, this association was not retained in the fully adjusted multivariate model (OR 1.15, 95% CI 0.60; 2.21) (Fig. 2). The supplementary exploratory analysis on the timing of pHC treatment yielded no significant association between alleviating pHC by tumour surgery alone and at 1, 2, 3 or ≥ 4 days prior to tumour surgery (Supplementary Table 9). A significant decrease in POSI risk was found between treating pHC 1 day and ≥ 4 days prior to tumour surgery (Supplementary Table 10).

**Table 4:** Odds ratio for postoperative speech impairment

Univariate analysis		Multivariate analyses		
		*Model 1 (tumour location)* *n* = *340*	*Model 2 (model 1* + *tumour type)* *n* = *312*	*Model 3 (model 2* + *age) n* = *312*
	*n* = *350*			
Preoperative hydrocephalus treatment *Reference: “no preoperative treatment”*^*a*^				
pHC treatment performed	1.93 (1.14; 3.26) *p* = *0.02*	1.33 (0.74; 2.39)	1.21 (0.63; 2.31)	1.15 (0.60; 2.21) *p* = *0.68*

Odds ratio results with 95% confidence intervals; *n* patients included in model; *pHC* preoperative hydrocephalus

^a^Preoperative hydrocephalus alleviated by tumour surgery alone

## Discussion

### Association between pHC and POSI

The most important finding of this study was the lack of association between pHC and the risk of POSI in the fully adjusted multivariate analysis. Our hypotheses were based on the assumption that periventricular oedema caused by elevated ICP could increase the risk of damage to critical neural structures involved in the development of POSI. The present findings support our previously published results, indicating that tumour type and location—rather than pHC itself—are likely to be the primary determinants of POSI. This suggests that any effect of pHC on POSI risk may be secondary to, or mediated by, tumour-related characteristics, indicating a more complex interaction than can be fully captured by the present analysis. Notably, we did not examine the association between pHC and other characteristics of CMS that impact quality of life, such as emotional disturbances or hypotonia.

To contextualize our findings, it is important to consider (1) the incidence of pHC in our cohort (64%), which corresponds to previously reported incidences [10, 17] and (2) the underlying mechanisms of pHC in PF tumours. Hydrocephalus can be obstructive, primarily depending on the location rather than the type of tumour and the most likely cause in our overall cohort. It can also be communicating, caused by impaired CSF reabsorption, evident by the increased risk of postoperative hydrocephalus requiring permanent CSF diversion in EP and MB patients [18, 19]. However, the pathophysiological mechanisms behind the latter aetiology have yet to be explored. Thus, the exact classification of pHC proved a challenge in the present cohort.

Although age was adjusted as a linear variable, uncertainty remains in assessing speech outcomes in very young children due to variation in early speech development. In our cohort, pHC incidence was relatively consistent across age groups, with no indication of an age-dependent effect on POSI risk. However, younger children may be more susceptible to pHC and its effects due to denser anatomy and a higher frequency of high-grade tumours [20].

### pHC treatment and POSI

pHC treatment prior to tumour surgery did not appear to influence speech outcomes. Combined with the results of the primary analysis, these findings overall imply that neither the presence of pHC nor its preoperative alleviation are independent risk factors for POSI. However, as practices differ between institutions, the timing of pHC treatment may be subject to further investigation to guide optimal management. The supplementary exploratory analysis revealed a trend toward treatment 1 to 2 days before surgery increasing POSI risk, while treatment 3 to ≥ 4 days prior reduced the risk, compared to pHC treated solely by tumour resection. These results may be suggestive of early hydrocephalus relief, shortly before tumour resection, increases the brain’s vulnerability to preoperative damage, while a longer interval between pHC treatment and tumour surgery facilitates stabilisation or reorganisation, reducing the fragility of critical structures related to speech. Nonetheless, it is important to recognise the following: (1) observed difference in POSI risk between the timing of pHC treatment may partly reflect the urgency, potentially serving as a proxy for the patient’s clinical condition, which could confound the results; (2) as both glucocorticoids (GC) and pHC treatment may reduce periventricular and parenchymal oedema, they can be seen as parallel strategies for preoperative stabilisation, potentially influencing the observed effect of each on POSI [21]. However, a recent publication from our group found no association between preoperative GC and POSI, and adjustment for preoperative GC, had the study been sufficiently powered, would likely not have altered our conclusions [22]. Finally, (3) pHC treatment concurrent with tumour resection was chosen as the reference due to its predominance in our cohort, making it the most representative baseline. This likely reflects the absence of standardisation in the timing of early tumour removal versus early pHC management, a discussion further complicated by conflicting evidence on the risk of persistent hydrocephalus. Some studies advocate for early pHC treatment to mitigate this risk [23, 24], while another study shows that tumour resection alone may suffice [25]. The lack of consensus on an optimal approach often leads clinicians to rely on familiar strategies, potentially explaining why alleviating pHC via tumour resection was the most prevalent strategy in our cohort. Additionally, certain tumour types may require earlier or more aggressive hydrocephalus management to address non-POSI-related complications, further challenging standardisation efforts. Given the limitations in clinical and surgical details in our study, this highlights the need for systematic research on the timing of pHC treatment across different tumour subgroups, as well as for standardised protocols.

Results from this study also reflect that clinical decisions regarding the timing of pHC intervention are individualised. We need to acknowledge the considerable ethical and logistical challenges in randomising the timing of pHC treatment in children undergoing primary PF tumour resection.

### Clinical implications

The lack of a significant association of pHC and its preoperative treatment with POSI in the multivariate analysis, along with the association of POSI with tumour pathology and location, are indicative of multifactorial mechanisms in its pathogenesis. Nevertheless, while the exploratory analysis suggests that early treatment of pHC may play a role in reducing the risk of POSI, it remains critical for reducing mortality and neurological dysfunction [10, 26]. Our findings should not warrant immediate changes to current clinical practice; early management of pHC continues to be essential for stabilising patients and ensuring appropriate surgical planning, even if its direct impact on speech outcomes remains somewhat uncertain.

### Impact of tumour pathology on the association between pHC and POSI

Our study observed a tumour-dependent trend in the impact of pHC on POSI, with a considerable decrease in the risk of POSI when pHC was not present in patients with MB and EP, although these findings were non-significant. Tumour pathology likely influences the association between pHC and POSI by impacting the rapidity and severity of hydrocephalus, thereby affecting speech-critical structures in the PF. In our study, MB was independently associated with a higher risk of POSI, aligning with a prior systematic review of 2276 children with PF tumours [1]. EP and MB frequently cause obstructive pHC due to their common location in the fourth ventricle, a recognised risk factor for POSI [4]. Moreover, up to 40% of MB cases exhibit leptomeningeal seeding, which can impair CSF reabsorption and cause persistent hydrocephalus, whereas the role of leptomeningeal spread in EP remains less defined [27]. By contrast, less aggressive tumours like PA, commonly located in the cerebellar hemisphere, are not as often linked to pHC and POSI due to their non-malignant features and typical location.

Although MB biologically involves heterogeneous tumours, we did not stratify our analyses by molecular subtype. While it is plausible that subtype-specific growth patterns and commonly associated anatomical locations could influence pHC development, our study was not powered to explore this hypothesis. To further address the impact of tumour pathology on the association between pHC and POSI, our results need to be verified in a setup that includes a larger prospective cohort. However, given the sample size of this study, it is unlikely that a future comparable setup will detect a strong interaction between pHC and tumour type on the risk of POSI.

### Limitations

We did not have an a priori definition of pHC in our study and instead relied on the neurosurgeons’ clinical and neuroradiological assessments. Including MRI measures like FOHR could have added objectivity, but the study would still be faced with individual clinical assessment and the corresponding treatment decisions, which formed the basis for the study’s prospective data registration. The study was therefore not designed to assess decision-making differences between neurosurgeons; however, the multicentric setup provides some degree of external validity to the findings and supports the generalisability to broader clinical settings.

Missing data may have influenced our findings; 12% were excluded from the primary and 35% from the secondary analysis due to incomplete data on POSI and pHC, potentially introducing selection bias. However, as no noteworthy differences in baseline characteristics were observed between the full cohort and the subcohorts of the primary and secondary analyses, we considered these to be broadly representative of the overall population.

Another limitation was the subjective assessment of POSI by clinicians. Despite consensus on POSI, the definition of reduced speech is open to interpretation, which may introduce inter-observer variability. A validated scoring tool could mitigate this bias, but no such tool was utilized in this study. POSI likely exists on a spectrum rather than as an ordinal outcome, and subtle impairments in speech may be missed. Previously reported word-finding difficulties in PF tumour patients [15, 28], further underscore the need for a more nuanced approach. Finally, this study did not examine long-term speech outcomes, which limits insight into the impact of pHC on speech recovery.

## Conclusion

The presence of pHC was not independently associated with POSI, nor did preoperative treatment of pHC prior to tumour resection appear to reduce the risk of POSI. These findings highlight the importance of individualizing pHC management in paediatric PF tumour cases, with decisions guided by the clinical context.

## Supplementary Information

Below is the link to the electronic supplementary material.

ESM1 (PDF 683 KB)

## Data Availability

The dataset generated and analysed in the current study is not publicly available due to ongoing recruitment. Data sharing will be considered upon reasonable request after study completion. Final follow-up of the last recruited participant is expected by the end of 2028.

## Abbreviations

AT/RT: Atypical teratoid/rhabdoid tumour
CI: Confidence interval
CMS: Cerebellar mutism syndrome
CSF: Cerebrospinal fluid
EP: Ependymoma
FOHR: Frontal-occipital horn ratio
GC: Glucocorticoids
ICP: Intracranial pressure
MB: Medulloblastoma
OR: Odds ratio
PA: Pilocytic astrocytoma
pHC: Preoperative hydrocephalus
PF: Posterior fossa
POA: Proportional odds assumption
POSI: Postoperative speech impairment
