# Supplementary material for: Preoperative hydrocephalus and the risk of postoperative speech impairment following posterior fossa tumour surgery in children: results from a prospective, multinational cohort study
Source: Childs Nerv Syst. 2026 Feb 4;42(1):60. doi: 10.1007/s00381-026-07132-z (PMC12872776; doi:10.1007/s00381-026-07132-z)
Supplement: Supplementary file 1 — (PDF 683 KB) [file 381_2026_7132_MOESM1_ESM.pdf]

Supplementary material

Title

Preoperative hydrocephalus and the risk of postoperative speech impairment following posterior fossa tumour surgery in children: results from a prospective, multinational cohort study

Authors

Aske Foldbjerg Laustsen, Radek Frič, Jonathan Kjær Grønbæk, Vladimír Beneš, Vicente Santa-Maria Lopez, Ulf Nestler, Andrea Carai, Guirish Solanki, Shivaram Avula, Conor Malluci, Pelle Nilsson, Per Nyman, Magnus Aasved Hjort, Rick Brandsma, Eelco Hoving, Antonella Bua, Jana Táborská, Katalin Mudra, Balazs Markia, Giedre Rutkaiskiene, Saulius Ročka, Jurgen Lemiere, Florian Wilhelmy, Christian Dorfer, Astrid Sehested, Marianne Juhler, René Mathiasen

Corresponding author

Aske Foldbjerg Laustsen (aske.foldbjerg.laustsen@regionh.dk)

Affiliation of corresponding author

Department of Neurosurgery, Rigshospitalet, Copenhagen, Denmark
Department of Pediatrics and Adolescent Medicine, Rigshospitalet, Copenhagen, Denmark

Supplementary figure 1 Flow diagram for registering preoperative hydrocephalus and treatment.

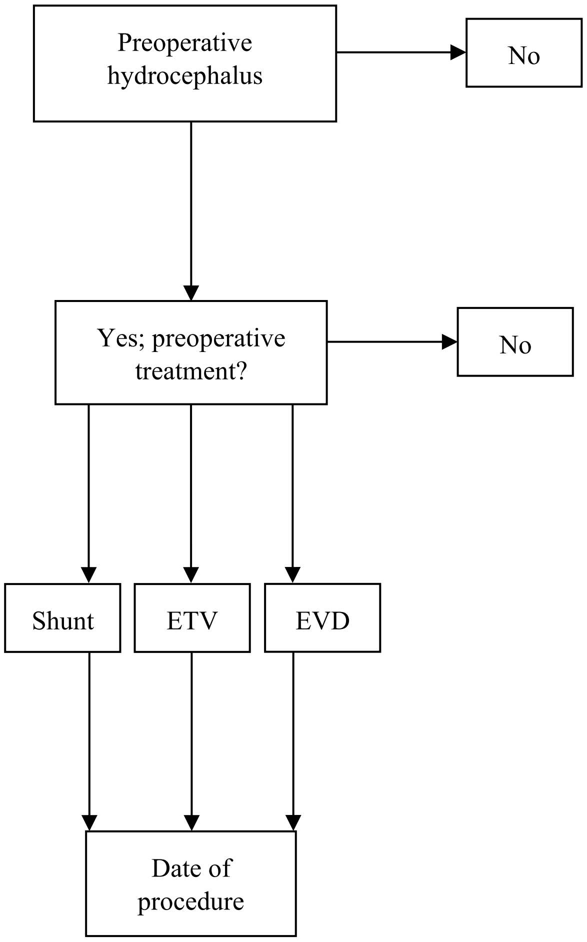

Supplementary table 1 “Other” tumour types as registered by the neurosurgeon or paediatrician

**Tumour classification included under ”other”-category**	**Amount (n = 73)**
Ganglioglioma	11
Choroid plexus papilloma	8
Subclassification not available	8
Diffuse midline glioma	6
Hemangioblastoma	6
Schwannoma	5
Low grade glioma without further specification	5
Embryonal tumour with multilayered rosettes	4
High grade glioma without further specification	4
Glioblastoma	3
Meningioma	2
Epidermoid cyst	2
Pineoblastoma	1
Mixed glioneuronal tumour	1
Astroglioma without further specification	1
Germ cell tumour without further specification	1
Gangliocytoma	1
Choroid plexus carcinoma	1
Giant cell osteoclastic tumour	1
Pilomyxoid astrocytoma	1
No tumour found	1

Supplementary table 2 Patients with pHC > 14 days from tumour surgery excluded from the secondary analysis.

pHC treatment ≥14 days prior to tumour surgery	All patients	Reduced speech	Mutism
41 days	1	0	0
49 days	1	0	0
552 days	1	0	0
1084 days	1	0	1
pHC preoperative hydrocephalus,			

Supplementary table 3 Brant test for POSI with preoperative hydrocephalus (yes/no)

**Model**	**Variable**	**X^2**	**df**	**Probability**
Univariate	Preoperative hydrocephalus	2.39	1	0.12
Model 1	Overall modelfit	7.71	7	0.36
	Preoperative hydrocephalus	1.11	1	0.29
Model 2	Overall modelfit	9.92	12	0.62
	Preoperative hydrocephalus	1.74	1	0.19
Model 3	Overall modelfit	10.02	13	0.68
	Preoperative hydrocephalus	1.63	1	0.20

Supplementary table 4 Brant test for POSI with preoperative hydrocephalus treatment (yes/no)

**Model**	**Variable**	**X^2**	**df**	**Probability**
Univariate	Preoperative hydrocephalus	0.03	1	0.86
Model 1	Overall modelfit	0.29	4	0.99
	Preoperative hydrocephalus	0.18	1	0.67
Model 2	Overall modelfit	4.99	8	0.76
	Preoperative hydrocephalus	0.26	1	0.61
Model 3	Overall modelfit	4.82	9	0.85
	Preoperative hydrocephalus	0.25	1	0.62

Supplementary table 5 Cohort characteristics based on available postoperative speech status (primary analysis)

	**All patients** (n=800); N (%)	**Known speech status** (n=727 (91%)); N (%)	**Missing speech status** (n=73 (9%)); N (%)
**Sex**			
Male	456 (57)	412 (57)	44 (60)
Female	344 (43)	315 (43)	29 (40)
**Age** (Years; Median and IQR)	6.9 (3.9;10.9)	7.0 (4.0;10.9)	6.0 (2.1;10.9)
**Tumour location**[a]			
Brainstem	156 (20)	142 (20)	14 (19)
4th ventricle	265 (33)	240 (33)	25 (34)
Vermis	141 (18)	129 (18)	12 (17)
Cerebellar hemisphere	187 (23)	176 (24)	11 (15)
Unknown	51 (6)	40 (5)	11 (15)
**Tumour histology**			
Pilocytic astrocytoma	305 (38)	297 (41)	8 (11)
Medulloblastoma	222 (28)	210 (29)	12 (16)
Ependymoma	79 (10)	73 (10)	6 (8)
AT/RT	20 (2)	17 (2)	3 (4)
Other	73 (9)	71 (10)	2 (3)
Unknown	101 (13)	59 (8)	42 (58)
**Preoperative hydrocephalus**			
Yes	515 (64)	472 (65)	43 (59)
No	240 (30)	220 (30)	20 (27)
Unknown	45 (6)	35 (5)	10 (14)
**Treatment for preoperative hydrocephalus prior to tumour surgery**			
	**n=515**	**n=472**	**n=43**
No	291 (58)	274 (58)	17 (40)
Yes	87 (17)	76 (17)	11 (25)
1[a]	19 (4)	17 (4)	2 (5)
2[a]	16 (3)	13 (3)	3 (7)
3[a]	21 (4)	20 (4)	1 (2)
≥4[a]	31 (6)	26 (6)	5 (11)
Unknown	137 (27)	122 (26)	15 (35)

Preoperative hydrocephalus subcohort used in the secondary and supplementary analyses, [a]Days prior to tumour resection

Supplementary table 6 Cohort and subcohort characteristics in secondary analysis

	**All patients** (n=800); N (%)	**Subcohort with pHC** (n=515 (64%)); N (%)	**Subcohort with registered date of pHC procedure** (n=378; N (%))
**Sex**			
Male	456 (57)	284 (55)	208 (55)
Female	344 (43)	231 (45)	170 (45)
**Age** (Years; Median and IQR)	6.9 (3.9;10.9)	6.6 (3.4;10.2)	6.6 (3.8;10.8)
**Tumour location**[a]			
Brainstem	156 (20)	93 (18)	58 (15)
4th ventricle	265 (33)	201 (39)	153 (40)
Vermis	141 (18)	98 (19)	75 (20)
Cerebellar hemisphere	187 (23)	105 (20)	82 (22)
Unknown	51 (6)	18 (4)	10 (3)
**Tumour histology**			
Pilocytic astrocytoma	305 (38)	174 (34)	140 (37)
Medulloblastoma	222 (28)	167 (32)	120 (32)
Ependymoma	79 (10)	58 (11)	40 (11)
AT/RT	20 (2)	16 (3)	6 (2)
Other	73 (9)	36 (7)	25 (7)
Unknown	101 (13)	64 (12)	47 (12)
**Postoperative speech impairment**			
Habitual	543 (68)	333 (65)	254 (67)
Reduced speech	96 (12)	68 (13)	51 (14)
Mutism	88 (11)	71 (14)	45 (12)
Unknown	73 (9)	43 (8)	28 (7)

Supplementary table 7 Odds Ratio of Postoperative Speech Impairment by preoperative hydrocephalus

**Univariate analysis**			**Multivariate analysis**			
			Model 1 (tumour location)		*Model 2 (model 1 + tumour type; n=616)*	*Model 3 (model 2 + age; n=614)*
	(*n=692*)	*Missing tumour location removed (n=666)*	*(n=666)*	*Missing tumour pathology removed (n=616)*		
**Preoperative Hydrocephalus**						
No	0.51 (0.35;0.76)	0.55 (0.37;0.82)	0.62 (0.41;0.95)	0.64 (0.42;0.98)	1.11 (0.56;2.21)	1.20 (0.60;2.41) *NS*
Yes	1 (ref)	1 (ref)	1 (ref)	1 (ref)	1 (ref)	1 (ref)
**Tumour location**						
Brainstem			0.87 (0.57;1.34)	0.90 (0.58;1.41)	1.05 (0.65;1.71)	1.06 (0.65;1.74) *NS*
4th ventricle			1 (ref)	1 (ref)	1 (ref)	1 (ref)
Vermis			0.26 (0.15;0.46)	0.24 (0.12;0.47)	0.34 (0.19;0.63)	0.34 (0.19;0.63) *p<0.0001*
Cerebellar hemisphere			0.14 (0.07;0.25)	0.09 (0.04;0.21)	0.18 (0.09;0.36)	0.19 (0.10; 0.36) *p<0.0001*
**Tumour pathology**						
PA					1 (ref)	1 (ref)
MB					2.45 (1.37;4.40)	2.35 (1.31;4.23) *p=0.004*
EP					2.01 (0.95;4.25)	1.59 (0.74;3.44) *NS*
AT/RT					3.66 (1.12;11.96)	2.34 (0.69;7.95) *NS*
Other					1.53 (0.62;3.77)	1.77 (0.70;4.45) *NS*
**Interaction between hydrocephalus and tumour pathology**						
pHC:PA					1 (ref)	1 (ref)
pHC:MB					0.61 (0.21;1.69)	0.58 (0.21;1.62) *NS*
pHC:EP					0.18 (0.03;1.03)	0.19 (0.03;1.06) *NS*
pHC:AT/RT					1.04 (0.09;11.59)	1.05 (0.09;11.98) *NS*
pHC/Other					0.50 (0.12;2.10)	0.52 (0.12;2.21) *NS*
**Age (per 1 year change)**						0.92 (0.88;0.97) *p=0.003*
Odds ratio results with 95% confidence intervals, *PA* Pilocytic Astrocytoma, *MB* Medulloblastoma, *EP* Ependymoma, *AT/RT* Atypical Teratoid/Rhabdoid Tumour, *pHC* preoperative hydrocephalus, *NS* not significant						

Supplementary table 8 Odds Ratio of Postoperative Speech Impairment by preoperative hydrocephalus treatment

Univariate analysis			Multivariate analysis			
			Model 1 (tumour location)		*Model 2 (model 1 + tumour type; n=312)*	*Model 3 (model 2 + age; n=312)*
	(*n=350*)	*Missing tumour location removed (n=340)*	*(n=340)*	*Missing tumour pathology removed (n=312)*		
**Preoperative Hydrocephalus treatment performed**						
Yes	1.93 (1.14;3.26)	1.76 (1.02;3.04)	1.33 (0.74;2.39)	1.18 (0.62;2.23)	1.21 (0.63;2.31)	1.15 (0.60;2.21) *NS*
No	1 (ref)	1 (ref)	1 (ref)	1 (ref)	1 (ref)	1 (ref)
**Tumour location**						
Brainstem			1.46 (0.79;2.70)	1.66 (0.87;3.17)	1.85 (0.93;3.68)	1.84 (0.92;3.67) *NS*
4th ventricle			1 (ref)	1 (ref)	1 (ref)	1 (ref)
Vermis			0.24 (0.12;0.47)	0.26 (0.12;0.57)	0.31 (0.14;0.73)	0.30 (0.13;0.69) *0.005*
Cerebellar hemisphere			0.07 (0.02;0.22)	0.07 (0.02;0.24)	0.09 (0.03;0.31)	0.09 (0.03;0.33) *p<0.001*
**Tumour pathology**						
PA					1 (ref)	1 (ref)
MB					1.83 (0.91;3.68)	1.75 (0.86;3.53) *NS*
EP					1.15 (0.46;2.89)	0.93 (0.36;2.39) *NS*
AT/RT					1.78 (0.27;11.63)	1.18 (0.17;7.99) *NS*
Other					1.91 (0.67;5.40)	2.20 (0.76;6.38) *NS*
**Age (per 1 year change)**						0.93 (0.87;1.00) *p=0.05*
Odds ratio results with 95% confidence intervals, *PA* Pilocytic Astrocytoma, *MB* Medulloblastoma, *EP* Ependymoma, *AT/RT* Atypical Teratoid/Rhabdoid Tumour, *pHC* preoperative hydrocephalus, *NS* not significant						

Supplementary table 9 Odds ratio of Postoperative Speech Impairment by preoperative treatment of hydrocephalus

**Odds ratio for Postoperative Speech Impairment**					
**Univariate analysis** (*n=350*)		**Multivariate analyses**			
		Model 1 (Tumour location) n=340	*Model 2 (Model 1 + tumour type) n=312*	*Model 3 (Model 2 + age) n=312*	
**Preoperative hydrocephalus treatment** (days before tumour surgery) *Reference: None (pHC alleviated by primary tumour surgery)*					
1	3.10 (1.24;7.74) *p=0.016*	2.25 (0.80;6.28)	2.31 (0.81;6.66)	2.15 (0.74;6.27) *p=0.16*	
2	2.05 (0.66;6.34) *p=0.21*	1.78 (0.52;6.05)	2.47 (0.59;10.40)	2.31 (0.55;9.81) *p=0.26*	
3	2.22 (0.89;5.57) *p=0.09*	1.19 (0.42;3.35)	0.82 (0.23;2.77)	0.76 (0.22;2.58) *p=0.66*	
≥4	1.13 (0.46;2.77) *p=0.79*	0.82 (0.31;2.18)	0.63 (0.20;1.98)	0.65 (0.21;2.03) *p=0.46*	
Odds ratio results with 95% confidence intervals, *pHC* preoperative hydrocephalus; *n* patients included in model					

Supplementary table 10 Odds ratio difference preoperative hydrocephalus treatment between 1 and ≥4 days

**OR for POSI** (difference between timing of pHC treatment between 1 day and ≥4 days)	**Model** (tumour location + tumour type + age) *OR (95 % CI)*
≥4 days (reference 1 day)	0.16 (0.03;0.90, *p=0.048*)

Supplementary table 11 Brant test for POSI with preoperative hydrocephalus treatment ("none", 1 day, 2 days, 3 days, 4 or more days)

	**Variable**	**X^2**	**df**	**Probability**
Univariate	Overall modelfit	1.15	4	0.89
	1 day	0.36	1	0.55
	2 days	0.21	1	0.65
	3 days	0.46	1	0.50
	4 or more days	0.02	1	0.89
Model 1	Overall modelfit	0.65	7	0.99
	1 day	0.39	1	0.53
	2 days	0.00	1	0.99
	3 days	0.00	1	0.99
	4 or more days	0.20	1	0.66
Model 2	Overall modelfit	6.69	11	0.82
	1 day	0.55	1	0.46
	2 days	0.30	1	0.58
	3 days	0.11	1	0.74
	4 or more days	1.24	1	0.26
Model 3	Overall modelfit	6.54	12	0.89
	1 day	0.58	1	0.45
	2 days	0.27	1	0.60
	3 days	0.11	1	0.74
	4 or more days	1.20	1	0.27
